# Behind the Therapeutic Effects of Royal Jelly: Recent Advances in the Specific Properties of 10-Hydroxydecanoic Acid

**DOI:** 10.3390/molecules30132694

**Published:** 2025-06-22

**Authors:** Carla Gasbarri, Guido Angelini

**Affiliations:** Department of Pharmacy, University “G. d’Annunzio” of Chieti-Pescara, 66100 Chieti, Italy; guido.angelini@unich.it

**Keywords:** Royal Jelly, 10-hydroxydecanoic acid, medium-chain fatty acid, bioactive compound, wound healing, anti-infective activity, hydrogel, dry eye treatment, docking simulation

## Abstract

Since ancient times, Royal Jelly (RJ) has been known for its remarkable properties in traditional medicine, and it is still widely recommended for mental and physical well-being. RJ consists of a unique and complex mixture of multiple constituents in different concentrations, and some of its biological activities are directly associated with specific components not found elsewhere in nature, such as (*E*)-10-hydroxy-2-decenoic acid (10-HDA) and its precursor 10-hydroxydecanoic acid (10-HDAA), two medium-chain fatty acids. Together, 10-HAD and 10-HDAA represent the major constituents of the total lipid fraction in RJ, but despite their structural similarity, the former has been extensively investigated over the years, while the latter has been only marginally reported. This review focuses on the promising effects of 10-HDAA that have emerged in a series of recent in vitro, in vivo, and docking simulation studies. Important bioactivities were observed for 10-HDAA, tested both as an individual compound, especially for immunoregulatory, estrogenic, and anti-inflammatory activities, and in synergic combination with other molecules. Specific anti-infective effects against endemic diseases, as well as the structural modification to synthesize biocompatible and biodegradable 10-HDAA-based amphiphiles, are also reported.

## 1. Introduction

Royal Jelly (RJ) is a natural yellowish creamy mixture slightly acidulous in taste, traditionally known for its myriad of nutritive, antioxidant, anti-inflammatory, immunomodulatory, and antimicrobial activities. Consequently, it is often recommended to people of all ages as a natural and functional supplement, both for physical weakness due to bacterial infections or intense pharmacological therapies and for mental stress due to anxiety and depression [1,2,3,4,5,6]. Furthermore, Royal Jelly-enriched products are commercially found in cosmetics for skin and hair care [7,8,9].

**Figure 1:**
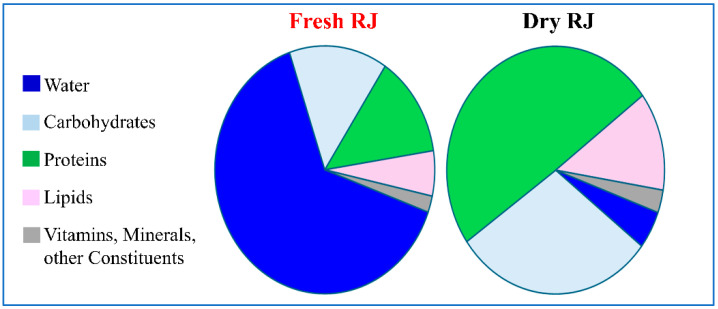
A comparison of the composition of fresh and dry Royal Jelly. The average percentages of the major constituents are shown. Data are from [5].

RJ is secreted by worker honeybees and mainly reserved as a source of nutrition for larvae for a few days and for the Queen bee throughout her entire life. It consists of a complex mixture mainly composed of water, carbohydrates, proteins, and lipids. RJ also contains vitamins, minerals, and other bioactive constituents, including amino acids, waxes, flavonoids, phenols, and sterols in small quantities [10]. In addition, acetylcholine, estradiol, prolactin, and testosterone have been identified [11]. It was observed that the lyophilization process extends the shelf life of Royal Jelly and improves its storage stability at room temperature. Interestingly, similar organoleptic features were detected in fresh and dry RJ, which is surprising considering that several chemical constituents are lost during freeze-drying [12,13]. A comparison of the composition of fresh and dry RJ is shown in Figure 1.

Although the chemical composition of RJ seems to be dependent on external factors, such as botanical origin, geographical location, environmental changes, and beehive conditions, such as the physiological state of the colony and production period, free fatty acids consistently represent the majority of its lipid fraction. In particular, RJ fatty acids are mainly medium-chain, containing 8 to 10 carbon atoms, saturated or monounsaturated at the 2-position. About 70% of the total fatty acids correspond to (*E*)-10-hydroxy-2-decenoic acid (10-HDA), commonly known as the Queen bee acid. The 10-HDA molecule is an alpha, beta-unsaturated carboxylic acid with a terminal hydroxylated 10-carbon chain. Due to its exclusive presence and its concentration in the lipid portion, the 10-HDA level is commonly measured to test the purity of Royal Jelly. Moreover, 10-HDA represents the standard marker to guarantee RJ quality against the risk of adulteration [14,15]. The Queen bee acid can be considered a natural bioactive compound since it has often been directly associated with the most therapeutic and pharmacological properties of RJ, especially its estrogenic, neurotrophic, anti-tumoral, and glucose-regulating effects [16,17,18,19]. Additionally, its specific role in photoaging and skin protection has been investigated [20,21].

The second component of the lipid fraction identified in RJ is 10-hydroxydecanoic acid (10-HDAA), accounting for 13–17% of total free fatty acids, followed by a series of minor constituents, such as 1,10-decanedioic acid (commonly referred to as sebacic acid), 8-hydroxyoctanoic acid, 3-hydroxydecanoic acid, 3,10-dihydroxydecanoic acid, 9-hydroxy-2-decenoic acid, and 2-decenedioic acid [22]. Interestingly, some studies suggest that the antioxidant and anti-inflammatory properties of Royal Jelly could be dependent on the position of the hydroxy and carboxylic groups in the fatty acid carbon chain [23].

Despite the strong similarity in their chemical structures, 10-HDA has attracted much more attention than 10-HDAA. Recently, Gao and co-workers [24] demonstrated that 10-HDA decreases the biofilm viability of *Staphylococcus aureus*. Sha et al. [25] reported its ability to maintain the fluidity of the erythrocyte membrane in the case of oxidative stress of the vascular smooth muscle cells. Lin and colleagues [26] reported the anti-tumor effects of 10-HDA in human lung cancer cells, and Fan et al. [17] reported a reduction in vascular smooth muscle cell inflammation. Many other remarkable studies could be cited as examples.

**Figure 2:**
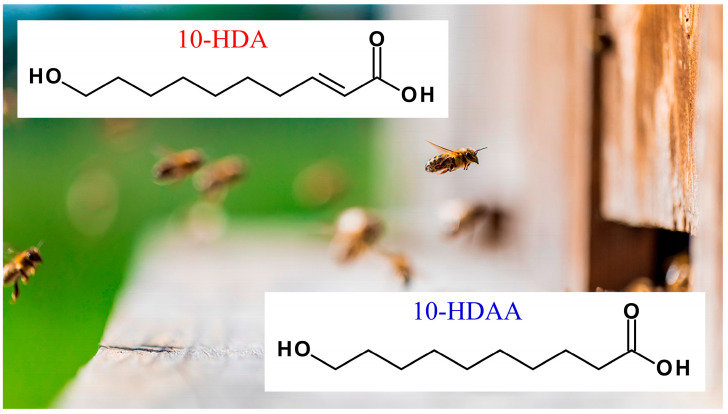
Chemical structures of (*E*)-10-hydroxy-2-decenoic acid (10-HDA) and 10-hydroxydecanoic acid (10-HDAA).

In addition, it has been observed that the 10-HDA molecule is fundamental for the development of larvae and the longevity of the Queen bee, but only the 10-HDAA molecule seems to be responsible for the immune-stimulating effect attributed to Royal Jelly, as described in Section 3.1. The molecular structures of 10-HDA and 10-HDAA are reported in Figure 2.

After their ingestion, both fatty acids are converted into sebacic acid via ω-oxidation. Dicarboxylic acids can be absorbed into the circulation and then excreted in the urine. The catabolism of sebacic acid produces succinyl-CoA and acetyl-CoA [27].

**Figure 3:**
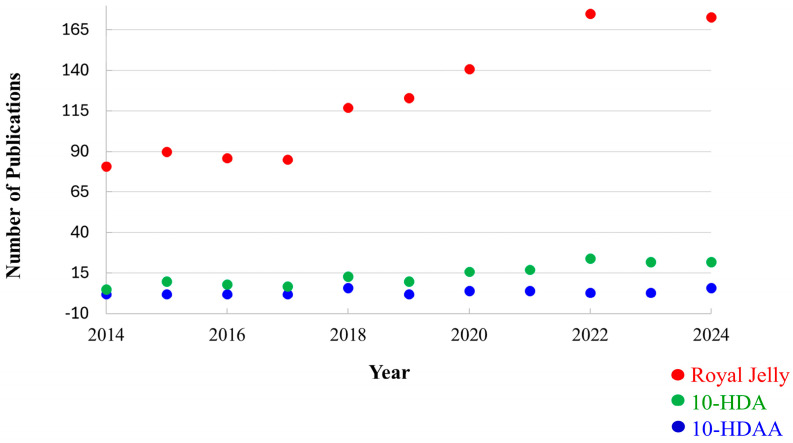
The number of documents obtained for the period 2014–2024 when searching the database Scopus for Royal Jelly, 10-hydroxy-2-decenoic acid, and 10-hydroxydecanoic acid.

The number of scientific publications focused on Royal Jelly increased from 81 in 2014 to 173 in 2024. As an example, Figure 3 shows the number of documents obtained for the period between 2014 and 2024 when searching the database Scopus for Royal Jelly, 10-hydroxy-2-decenoic acid, and 10-hydroxydecanoic acid. When the results are limited to the English language, the total number of documents is 1452, 154, and 36, respectively.

Although attention to Royal Jelly and its benefits has been growing in the last several decades, the number of documents focused on 10-HDAA is still limited. To the best of our knowledge, we report the most recent remarkable results on 10-HDAA from a series of studies to highlight its potential effects as a natural bioactive compound, contributing to the development of novel materials and formulations for expanding the therapeutic applications of Royal Jelly and its precious individual constituents.

## 2. Extraction and Synthesis

The most common methods for isolating fatty acids from RJ are based on extraction using conventional organic solvents. Noda and co-workers isolated and analyzed the lipid fraction of RJ by using chloroform and chloroform/methanol mixtures. Each component was then identified by means of spectroscopical techniques. A yield of 3.2 mg of 10-HDAA was obtained from 1.5 kg of lyophilized RJ powder [28]. Soxhlet or homogeneous liquid–liquid extractions can also be performed. It was observed that the different extraction techniques employed for Royal Jelly offer a combination of advantages and drawbacks: the main limitations are solvent toxicity and long times in the case of solvent extraction, the risk of fatty acid degradation at high temperatures in the case of Soxhlet extraction, and the possible limitation of further applications of the extracted fatty acids due to the solvents and dispersants employed in the case of homogeneous liquid–liquid extraction [22]. High levels of efficiency and sustainability, fast times, and better yields were recently described using ultrasound- and microwave-assisted techniques, supercritical fluid extraction, pulsed electric field-assisted extraction, and high-pressure-assisted extraction [29]. It was demonstrated that the ultrasound-assisted extraction of fatty acids from lyophilized RJ previously dissolved in ethanol and then sonicated provided 16.89% (±0.10) 10-HDAA, in comparison to the 16.91% (±0.08) obtained by solvent extraction [30]. A synthetic approach to obtaining 10-HDAA and other bioactive constituents from Royal Jelly was also proposed as an alternative to extraction. The microwave-assisted synthesis of 10-HDAA was performed by Zhang and Gao [31], starting with castor oil as the raw material according to a green approach. A process that reduced the time by about 2/3 in comparison to the classical heating treatment was carried out, leading to a yield of above 77% for 10-HDAA using 167 °C as the reaction temperature, 120 min as the reaction time, and 1/1.25/1.5 as the castor oil/NaOH/*sec*-octanol ratio.

In other studies, castor oil was replaced with sodium ricinoleate to use only reagents in the solid state, thereby avoiding the addition of thinning agents and allowing solid-phase cleavage. However, this process is generally carried out for the industrial production of 1,10-decanedioic acid, generating 10-HDAA as a side product of the reaction [32]. Furthermore, the synthetic derivatives of Royal Jelly are generally not food-grade and are commonly excluded from food and health products for safety reasons [33]. A biocompatible and successful method to synthesize 10-HDAA from natural sources and increase its availability was recently proposed by Itatani et al. The natural content of 10-HDAA in Royal Jelly was enriched about five times due to fermentation induced by bacteria isolated from the digestive tract of Queen bees which promotes the conversion of 10-HAD into 10-HDAA [34]. The fermentation process was conducted under anaerobic conditions and was completed in 5 days. The resulting biotreated RJ was developed as a potential immune-stimulating food in light of the promising activity of 10-HDAA on antigen-specific IgA expression, as described in the next section.

## 3. Biological Activities

### 3.1. Antigen-Specific Immune Response Efficacy

The Microfold cells, or M-cells, are intestinal epithelial cells specifically involved in mucosal immune responses through the uptake and transcytosis of luminal antigens [35]. Despite their fundamental role, the mechanisms by which M-cell function and differentiation occur are still under investigation.

**Figure 4:**
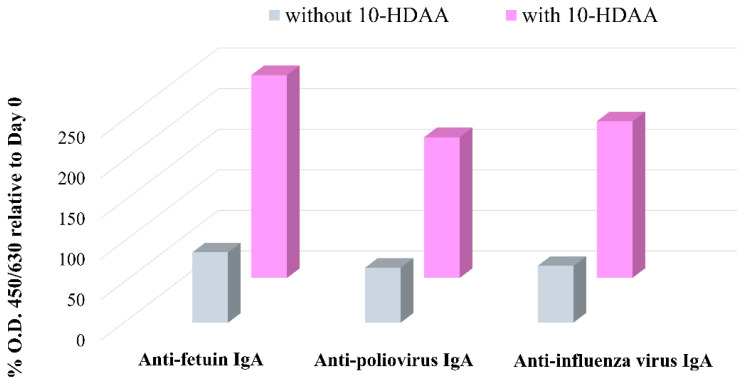
IgA response on day 12 against fetuin, poliovirus, and influenza virus with and without 10-HDAA. The % O.D. values are reported as the mean data from three macaques in the investigated conditions. Data are from [36].

The potential role of 10-HDAA in supporting Microfold cell differentiation and promoting an antigen-specific immune response was first reported by Misumi et al. [36]. Three different enteric-coated capsules, consisting of fetuin (a), inactivated antigens from poliovirus (b), and influenza virus (c), were orally administrated with or without 10-HDAA to cynomolgus macaques. The enhanced IgA level was monitored via stool samplings 12 and 21 days after administration by measuring the absorbance at 450 and 630 nm (O.D. 450/630). The data were expressed as percentage O.D. on days 12 and 21 relative to the value observed on day 0. The results indicate that the production of IgA in the conditions investigated was enhanced on day 12. Moreover, an antigen-specific mucosal response was strongly promoted by the 10-HDAA treatment in all cases. The data obtained on day 12 with and without 10-HDAA in the immunized macaques against fetuin, poliovirus, and influenza virus are reported in Figure 4.

Furthermore, an increase in the number of M-cells in the follicle-associated epithelium covering Peyer’s patches and in the epithelium overlying the nasopharynx-associated lymphoid tissue was observed in the macaques that received 10-HDAA via oral and intranasal administration.

### 3.2. Estrogenic Activities

The interaction between Royal Jelly and the estrogen receptors involved in gene expression and cell proliferation was comprehensively described by Mishima et al. determining the estrogenic activity of specific constituents isolated from RJ, including 10-HDAA [37]. The capability of the investigated compounds to bind to the estrogen receptors α (ERα) and β (ERβ) in competition with 17β-estradiol was determined and compared to diethylstilbestrol activity. A 10-HDAA yield of 0.53 g was obtained through extraction from 2000 g of fresh RJ and analyzed using NMR analysis after purification and recrystallization. It was observed that the 10-HDAA molecule and other selected compounds from RJ act as weak inhibitors of the binding of 17β-estradiol to ERβ in a concentration-dependent manner. In particular, IC_50_ values of 21 nM and 140 μM were measured for diethylstilbestrol and 10-HDAA, respectively. No inhibitory effect on the binding of 17β-estradiol to ERα was detected for 10-HDAA. Moreover, enhanced transcription following the transient transfection of MCF-7 cells with a reporter gene containing an estrogen-responsive element suggested that in the investigated conditions, 10-HDAA activated ERs, leading to gene expression and cell proliferation.

### 3.3. Anti-Inflammatory Properties

**Figure 5:**
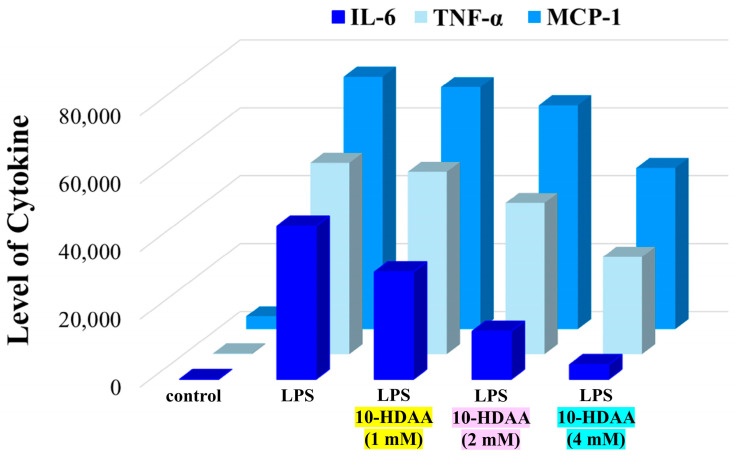
Comparison of IL-6, TNF-α, and MCP-1 levels in BV-2 cells after LPS-induced inflammation and 10-HDAA pretreatment at different concentrations. Data are from [40].

Previous studies focused on the anti-inflammatory effects of the major RJ fatty acids in vitro showed that 10-HDAA exhibits remarkable and dose-dependent inhibitory activities on interleukin-10 release, nitric oxide production, and inducible nitric oxide synthase mRNA expression during the inflammatory process in macrophages induced by lipopolysaccharide treatment [38]. Furthermore, the negligible effect on interleukin-6 release and the lack of inhibitory activity against TNF-α, both of which are crucial inflammatory mediators, suggest that 10-HDAA could act on the mRNA expression of target genes. Lipopolysaccharide (LPS) is commonly used to mimic the inflammation process in neurodegenerative diseases [39]. More recently, the effects of 10-hydroxydecanoic acid on microglial cells under LPS-induced inflammation were investigated [40]. Microglia are specific types of macrophages of the central nervous system and are involved in the development, maintenance, and injury repair of neuronal networks [41]. Any alteration or overactivation could be responsible for acute or chronic neurodegenerative disease [42]. It was observed that pretreatment with 10-HDAA reduces the levels of nitric oxide and inducible nitric oxide synthase in microglial cells activated by the bacterial endotoxin lipopolysaccharide. In particular, 10-HDAA suppressed the increase in interleukin-6 (IL-6), TNF-α, and monocyte chemotactic protein (MCP-1) levels in a concentration-dependent manner. Similar results were obtained for two microglial cell lines (BV-2 and N9). The quantification of the cytokines IL-6, TNF-α, and MCP-1 in the culture medium of a BV-2 cell array determined by using cytometric beads under the investigated conditions is reported in Figure 5 as an example. Interestingly, it was also demonstrated that the tumor suppressor p53 mediates the anti-neuroinflammatory effect, acting as a target for 10-HDAA.

The same team of researchers also demonstrated that, when combined, 10-HDAA and aspirin have synergistic effects against LPS-induced neuroinflammation [43]. Moreover, promising results, such as the inhibition of the overactivation of glial cells, a decrease in the levels of pro-inflammatory mediators, and the relief of side effects of aspirin on the gastrointestinal tract and microbiota dysbiosis, suggest that the investigated combination based on 10-HDAA as a natural bioactive compound could be considered as an innovative therapeutic strategy for treating inflammation-related neurodegenerative diseases.

Another relevant case of synergism was demonstrated for the association between the 10-HDAA molecule and zinc oxide nanoparticles (ZnONPs). The combined system was tested against testicular and renal toxicity induced in rats via oral administration of lead acetate for 3 months [44,45]. The data reported on oxidation, inflammation, and apoptosis after treatment with 10-HDAA and/or ZnONPs for 1 month revealed better efficacy with the 10-HDAA/ZnONP combination in comparison to the individual use of 10-HDAA or ZnONPs in monotherapy.

### 3.4. Anti-Infective Activities

Malaria and Leishmaniasis are endemic infections caused by *Plasmodium* and *Leishmania* parasites, respectively, in tropical and subtropical countries. In the last few decades, a series of adverse side effects and drug resistance have emerged with the use of conventional therapies in both diseases. Moreover, the possibility of co-infection due to geographical overlap was pointed out [46].

The antimalarial and antileishmanial activities of 10-hydroxydecanoic acid were tested and compared to Royal Jelly, 10-HDA, and sebacic acid activities [47]. The 50% inhibitory concentration (IC_50_) values were determined for each compound against the chloroquine-resistant *Plasmodium falciparum* K1 strain and against *Leishmania major* amastigotes. Interestingly, the results indicate a better efficacy for 10-HDAA as a single molecule in comparison to Royal Jelly. In addition, the 50% lethal concentration (LC_50_) value against *Aedes aegypti* larvae at 25 ± 2 °C after 24 h of incubation was measured to estimate the insecticidal activity of the investigated compounds. In all cases, LC_50_ values below 50 mg/L were obtained, suggesting a remarkable larvicidal effect [48,49]. In particular, an LC_50_ value of 37.8 μg/mL was measured for 10-HDAA in the experimental conditions. In the same study, remarkable data were also reported on cytotoxicity, nitric oxide production, plasma membrane permeability, and caspase 3-like levels in extract-treated promastigotes.

### 3.5. Wound Healing Activity

**Figure 6:**
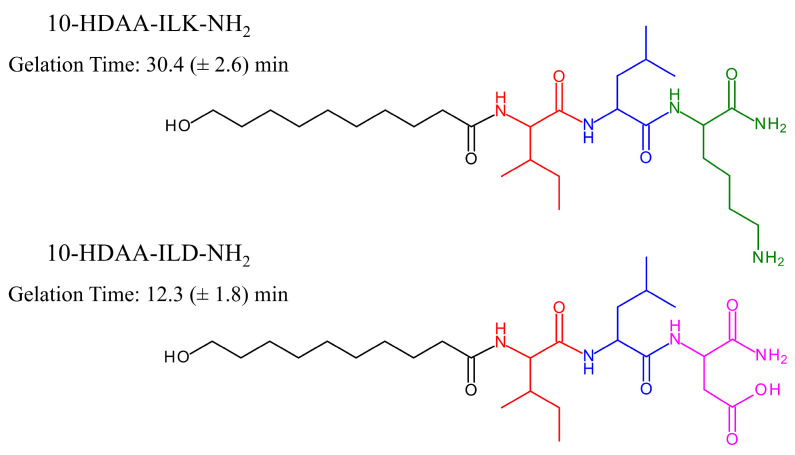
Chemical structures and gelation times of 10-HDAA-ILK-NH_2_ and 10-HDAA-ILD-NH_2_. The moieties of the single constituents are depicted in different colors as follows: black for 10-HDAA; red for isoleucine; blue for leucine; green for lysine; and pink for aspartic acid. Data are from [59].

Amphiphiles represent a well-known class of molecules containing both a polar headgroup and a lipophilic moiety and are able to self-assemble into a large variety of supramolecular structures in aqueous solution. The aggregation of amphiphilic molecules is a spontaneous process mainly based on hydrophobic forces, electrostatic interactions, and non-covalent bonding. The resulting supramolecular aggregates exhibit different sizes, morphology, stability, and rheological properties. Moreover, phase transitions can be triggered by changing the conditions under which aggregation occurs and may readily affect their use in delivery systems, technological devices, and pharmacological applications [50,51,52,53]. Peptides and natural amino-acid-based hydrogels show high versatility as therapeutic formulations, especially in wound healing treatments, due to their high biocompatibility and biodegradability [54,55]. Considering the efficacy of Royal Jelly as a natural agent for healing infected or chronic wounds and the specific contribution of its constituents to antimicrobial and tissue reparative activities [56,57,58], the preparation of hydrogels based on amino acids and 10-HDAA could provide novel bioactive materials. Hong and co-workers recently designed, synthesized, and investigated a class of novel amphiphiles obtained by covalently bonding the main RJ hydroxy fatty acids, including 10-HDAA, to specific tripeptides. The molecular modification of selected fatty acids from Royal Jelly was performed through solid-phase organic synthesis, with the aim of obtaining biodegradable, biocompatible, and non-toxic amphiphiles capable of forming hydrogels with wound repairing and antimicrobial activities [59]. The amino acids isoleucine (I), leucine (L), aspartic acid (D), and lysine (K) were linked by peptide bonds to hydroxy fatty acids, converting the C-terminus into the amidated form (NH_2_). The chemical structures of the synthesized amphiphiles 10-HDAA-ILK-NH_2_ and 10-HDAA-ILD-NH_2_ based on the 10-HDAA structure with their corresponding gelation times are reported in Figure 6.

The hydrogels formed by 10-HDAA-ILD-NH_2_ and 10-HDAA-ILK-NH_2_ in the investigated conditions are injectable, non-Newtonian fluids with shear-thinning behavior. Interestingly, the former demonstrates high stability in phosphate-buffered saline solutions at different pH values and the tendency to insert into wounds, while the latter exhibits a remarkable bacteriostatic effect against strains of *Staphylococcus aureus*. Such amphiphiles based on the molecular modification of 10-HDAA could find important applications as antibacterial agents in cases of infection and tissue regeneration.

### 3.6. Gene Expression Modulation Activity

The fundamental role of natural bioactive compounds in chemoprevention is well known and widely reported in the literature. Currently, most chemotherapeutic drugs are isolated from sustainable sources or provide molecular models to develop alternative chemopreventive agents [60,61,62].

An important contribution to emerging strategies based on gene expression modulation was recently reported by Rainho and co-workers [63]. Molecular docking simulations and biochemical assays were used to examine the modulation induced by different fatty acids of Royal Jelly on gene expression in mammalian cells. In particular, the inhibition of histone deacetylase enzymes (HDACs) was tested. The binding affinity energy values of the investigated molecules for the catalytic domain were calculated and compared both to phenylbutyrate, selected as a molecule associated with the capability to inhibit HDACs, and to dihydroferulic acid, selected as a molecule never associated before with HDAC inhibition. The binding affinity energy values determined for phenylbutyrate and dihydroferulic acid are −5.8 kcal/mol and −7.3 kcal/mol, respectively. Binding affinity energy values of −5.8 and −6.3 kcal/mol were reported for 10-HDAA and 10-HDA, respectively, suggesting similar HDAC-inhibitory behavior for the 10-HDAA and phenylbutyrate molecules. Furthermore, it was observed that, alone or combined, 10-HDAA and 10-HDA inhibit the activity of human nuclear HDACs, leading to a slight increase in the expression of HDAC-coding genes in cancer cells. Although both can be considered weak enzymatic inhibitors, durable effects were observed in the upregulated expression of target genes.

### 3.7. Ocular Treatment for Dry Eye Diseases

**Figure 7:**
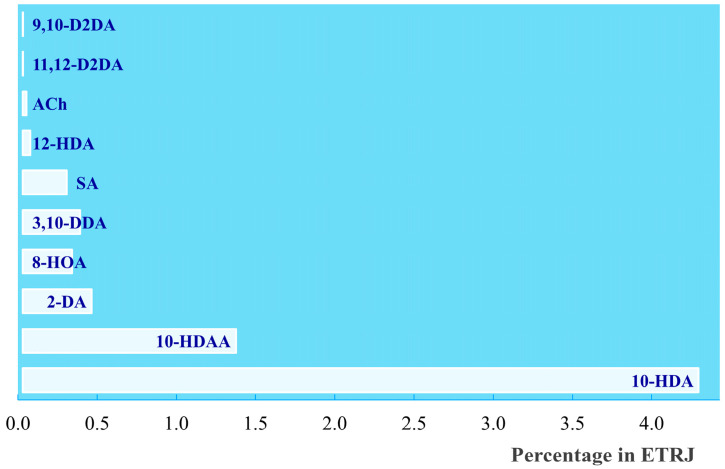
Percentage contents of the investigated components in ETRJ: 9,10-D2DA (9,10-dihydroxy-2-decenoic acid); 11,12-D2DA (11,12-dihydroxy-2-dodecenoic acid); ACh (Acetylcholine); 12-HAD (12-hydroxydodecanoic acid); SA (sebacic acid); 3,10-DDA (3,10-dydroxydecanoic acid); 8-HOA (8-hydroxyoctanoic acid); 2-DA (2-decenedioic acid); 10-HDAA (10-hydroxydecanoic acid); and 10-HDA (10-hydroxy-2-decenoic acid). Data are from [68].

The report of the Tear Film and Ocular Surface Society Dry Eye WorkShop (DEWS) Epidemiology subcommittee published in 2007 indicated that the prevalence of diseases involving dry eye in individuals over the age of 50 ranged from 5 to 30% [64]. In the second report, published in 2017, a further increase to 50% was determined [65]. It is highly likely that this percentage is still growing, considering that the primary causes of dry eye diseases are linked to the global use of smartphones, computers, and air conditioning. The functional decline in tear production seems to be related to the inflammation process promoted by oxidative stress [66], and for this reason, functional foods with anti-inflammatory and antioxidant activities may be recommended in some cases for ocular treatments. Interestingly, it was observed that the oral administration of Royal Jelly for 8 weeks can improve tear secretion in symptomatic patients affected by dry eye disease [67]. Tsubota and co-workers [68] thoroughly investigated the effect of RJ and its bioactive constituents on tear secretion by using a stress-induced dry eye mice model. In particular, acetylcholine and three fatty acids, including 10-HDAA, were tested individually or combined via oral administration to six mice for each experiment. Acetylcholine (ACh) is one of the bioactive components detected in RJ [11] and was previously associated with the contraction induced by oral administration of RJ on ileal smooth muscle, acting as a cholinergic neurotransmitter able to mediate the excitation of intestinal smooth muscle through M-receptors [69,70]. The active role of ACh from Royal Jelly on tear secretion was studied by using a hypoallergenic enzyme-treated RJ (ETRJ) powder [71] containing 0.023% ACh, 1.347% 10-HDAA, 4.267% 10-HDA, and seven other fatty acids in the range of 0.001–0.435%. The percentage amounts of the investigated bioactive components in 300 mg/kg of ETRJ are reported in Figure 7.

The data indicate that 10-HDAA, 8-HOA, and 3,10-DDA, in combination with ACh, are essential for tear secretion in the investigated ocular treatment. In particular, the results suggest that the three fatty acids from Royal Jelly could prevent the ACh degradation induced by ACh esterase.

## 4. Conclusions

Because of its nutritional and functional constituents and well-known beneficial and therapeutic properties, Royal Jelly can be considered a gift from nature. RJ is a creamy mixture secreted by the mandibular and hypopharyngeal glands of worker bees to feed all honeybee larvae for the first three days after birth and feed Queen bee larvae for the rest of their lives. Honey and RJ represent the most studied products from beehives due to their remarkable properties, including wound healing, anti-inflammatory, and immunomodulatory activities [72]. In addition, anti-aging effects and estrogen-like behavior have been identified in the case of RJ, mainly due to the unique presence of 10-HAD and 10-HDAA [37,73]. The versatility of honey and Royal Jelly has been widely demonstrated in the last several decades for a large series of applications, such as medical remedies, functional foods, and cosmetics. Besides their differences in composition and nutritional value, studies on honey used in its entirety, without extracting any individual components, tend to only mention its botanical source and geographical origin, while in the case of Royal Jelly, there is a tendency to link its biological activities to specific active constituents to more comprehensively understand the action mechanism and their individual properties. A large number of scientific articles revealed that the important effects of RJ may be directly attributed to 10-HDA and 10-HDAA.

This review highlights the biological activities of 10-HDAA as an individual molecule, in combination with other bioactive compounds, or compared to selected medium fatty chains from Royal Jelly. The results indicate its remarkable effects on gene expression and the immune response, as well as its estrogenic, anti-inflammatory, and anti-infective applications. The design and synthesis of the novel hydrogels formed by amphiphiles structurally based on the 10-HDAA molecule for wound healing treatment have also been reported. Furthermore, the use of Royal Jelly in the treatment of dry eye diseases has yielded promising data. The bioactive behavior of 10-HDAA is still under investigation, and all studies reported in this review confirm the strong versatility of the molecule as a promising natural compound for a large variety of applications in the biological, biochemical, and pharmacological fields.

## Data Availability

Not applicable.

## References

[1] Botezan S., Baci G.M., Bagameri L., Pașca C., Dezmirean D.S. (2023). Current Status of the Bioactive Properties of Royal Jelly: A Comprehensive Review with a Focus on Its Anticancer, Anti-Inflammatory, and Antioxidant Effects. Molecules.

[2] Pavel C.I., Mărghitaș L.A., Bobiș O., Dezmirean D.S., Şapcaliu A., Radoi I., Mădaș M.N. (2011). Biological activities of royal jelly-review. Sci. Pap. Anim. Sci. Biotech..

[3] Miryan M., Tadibi V., Sadeghi E., Najafi F., Saber A., Abbaspour M., Pasdar Y. (2025). The effect of royal jelly in oxidative stress, athletic performance, and mitochondrial biogenesis-related gene expression in endurance athletes: Study protocol for a double-blind crossover trial. Trials.

[4] Iegaki N., Narita Y., Hattori N., Hirata Y., Ichihara K. (2020). Royal jelly reduces depression-like behavior through possible effects on adrenal steroidogenesis in a murine model of unpredictable chronic mild stress. Biosci. Biotechnol. Biochem..

[5] Collazo N., Carpena M., Nunez-Estevez B., Otero P., Simal-Gandara J., Prieto M.A. (2021). Health promoting properties of bee royal jelly: Food of the queens. Nutrients.

[6] Ahmad S., Campos M.G., Fratini F., Altaye S.Z., Li J. (2020). New insights into the biological and pharmaceutical properties of royal jelly. Int. J. Mol. Sci..

[7] Dumitru C., Neacsu I., Grumezescu A., Andronescu E. (2022). Bee-derived products: Chemical composition and applications in skin tissue engineering. Pharmaceutics.

[8] Gatea A.H., Anatheil A.H., Ali A.M. (2023). Overview on Bee products in Skin care and Hair care. Mod. Med. Lab. J..

[9] Kawano Y., Makino K., Jinnin M., Sawamura S., Shimada S., Fukushima S., Ihn H. (2019). Royal jelly regulates the proliferation of humandermal microvascular endothelial cells through the down-regulation of a photoaging-related microRNA. Drug Discov. Ther..

[10] Alkindi F.K.S.A., El-Keblawy A., Ridouane F.L., Mirza S.B. (2024). Factors influencing the quality of Royal jelly and its components: A review. Cogent Food Agric..

[11] Guo J., Wang Z., Chen Y., Cao J., Tian W., Ma B., Dong Y.J. (2021). Active components and biological functions of royal jelly. J. Funct. Foods.

[12] Maghsoudlou A., Mahoonak A.S., Mohebodini H., Toldra F. (2019). Royal Jelly: Chemistry, Storage and Bioactivities. J. Apic. Sci..

[13] Kausar S., More V. (2019). Royal Jelly: Organoleptic Characteristics and Physicochemical Properties. Pharm. Chem. J..

[14] Ramadan M.F., Al-Ghamdi A. (2012). Bioactive compounds and health-promoting properties of royal jelly: A review. J. Funct. Foods.

[15] Sabatini A.G., Marcazzan G.L., Caboni M.F., Bogdanov S., de Almeida-Muradian L.B. (2009). Quality and standardisation of Royal Jelly. J. ApiProd. ApiMed. Sci..

[16] Moutsatsou P., Papoutsi Z., Kassi E., Heldring N., Zhao C., Tsiapara A., Melliou E., Chrousos G., Chinou I., Karshikoff A. (2010). Fatty acids derived from royal jelly are modulators of estrogen receptor functions. PLoS ONE.

[17] Jia F., Wang Y., Chen Z., Jin J., Zeng L., Zhang L., Tang H., Wang Y., Fan P. (2025). 10-Hydroxydec-2-enoic acid reduces vascular smooth muscle cell inflammation via interacting with Toll-like receptor 4. Phytomedicine.

[18] Gong Y., Luo H., Li Z., Feng Y., Liu Z., Chang J. (2023). Metabolic Profile of Alzheimer’s Disease: Is 10-Hydroxy-2-decenoic Acid a Pertinent Metabolic Adjuster?. Metabolites.

[19] Hu X., Liu Z., Lu Y., Chi X., Han K., Wang H., Wang Y., Ma L., Xu B. (2022). Glucose metabolism enhancement by 10-hydroxy 2-decenoic acid via the PI3K/AKT signaling pathway in high-fat-diet/streptozotocin induced type 2 diabetic mice. Food Funct..

[20] Park H.M., Hwang E., Lee K.G., Han S.M., Cho Y., Kim S.Y. (2011). Royal Jelly Protects Against Ultraviolet B–Induced Photoaging in Human Skin Fibroblasts via Enhancing Collagen Production. J. Med. Food.

[21] Maeda Y., Fujikura C., Asama T., Yagi M., Okumura N., Yamaki A., Ohkuma A., Numano K. (2022). Effect of Facial Application of Essence Containing Royal Jelly Extract on Stratum Corneum Moisture Content: A Placebo- Controlled, Double- Blind, Parallel-Group Study. J. Cosmet. Dermatol..

[22] Yu X., Tu X., Tao L., Daddam J., Li S., Hu F. (2023). Royal Jelly Fatty Acids: Chemical Composition, Extraction, Biological Activity, and Prospect. J. Funct. Foods.

[23] Melliou E., Chinou I. (2005). Chemistry and bioactivity of royal jelly from Greece. J. Agric. Food Chem..

[24] Gao K., Su B., Dai J., Li P., Wang R., Yang X. (2022). Anti-biofilm and Anti-hemolysis activities of 10-hydroxy-2-decenoic acid against Staphylococcus aureus. Molecules.

[25] Sha F., Yang P., Wang H., Ren J., Li Z., Zhang L., Fan P. (2023). 10-Hydroxydec-2- enoic acid enhances the erythrocyte membrane fluidity via interacting with phosphatidylcholine and phosphatidylethanolamine. Ital. J. Food Sci..

[26] Lin X.M., Liu S.B., Luo Y.H., Xu W.T., Zhang Y., Zhang T., Xue H., Zuo W.B., Li Y.N., Lu B.X. (2020). 10-HDA induces ROS-mediated apoptosis in A549 human lung cancer cells by regulating the MAPK, STAT3, NF-κB, and TGF-β1 signaling pathways. BioMed Res. Int..

[27] Yamaga M., Tani H., Yamaki A., Tatefuji T., Hashimoto K. (2019). Metabolism and pharmacokinetics of medium chain fatty acids after oral administration of royal jelly to healthy subjects. RSC Adv..

[28] Noda N., Umebayashi K., Takafumi N., Miyahara K., Ishiyama K. (2005). Isolation and Characterization of Some Hydroxy Fatty and Phosphoric Acid Esters of 10-Hydroxy-2-decenoic Acid from the Royal Jelly of Honeybees (*Apis mellifera*). Lipids.

[29] Wen L., Zhang Z.H., Sun D.W., Sivagnanam S.P., Tiwari B.K. (2020). Combination of emerging technologies for the extraction of bioactive compounds. Crit. Rev. Food Sci..

[30] Yu X., Li S., Peng S., Tao L., Hu F. (2024). Optimization of ultrasound-assisted extraction of fatty acids from royal jelly and its effect on the structural and antioxidant property. Ultrason. Sonochem..

[31] Zhang G.M., Gao H. (2008). Study on the green synthesis process of 10-hydroxydecanoic acid. Guangdong Chem..

[32] Yu S., Cui J., Zhong C., Meng J., Xue T. (2019). Green process without thinning agents for preparing sebacic acid via solid-phase cleavage. ACS Omega.

[33] Liao Z., Alrosan M., Aludatt M.H., Tan T.C. (2024). 10-hydroxy decanoic acid, trans-10-hydroxy-2-decanoic acid, and sebacic acid: Source, metabolism and potential health functionalities and nutraceutical applications. J. Food. Sci..

[34] Itatani H., Yamaki A., Konishi K., Okamoto H., Okumura N., Shigematsu N., Misumi S., Takenaka S. (2025). Fermented Royal Jelly Enriched With 10-Hydroxydecanoic Acid and Its Potential for Enhancing Mucosal Immunity. Food Sci. Nutr..

[35] Kanaya T., Ohno H. (2014). The Mechanisms of M-cell Differentiation. Biosci. Microbiota Food Health.

[36] Isayama T., Etoh H., Kishimoto N., Takasaki T., Kuratani A., Ikuta T., Tatefuji T., Takamune N., Muneoka A., Takahashi Y. (2020). 10-Hydroxydecanoic Acid Potentially Elicits Antigen- Specific Iga Responses. Biol. Pharm. Bull..

[37] Suzuki K.M., Isohama Y., Maruyama H., Yamada Y., Narita Y., Ohta S., Araki Y., Miyata T., Mishima S. (2008). Estrogenic activities of Fatty acids and a sterol isolated from royal jelly. Evid. Based Complement. Alternat. Med..

[38] Chen Y.F., Wang K., Zhang Y.Z., Zheng Y.F., Hu F.L. (2016). In Vitro Anti-Inflammatory Effects of Three Fatty Acids from Royal Jelly. Mediat. Inflamm..

[39] Qin L., Wu X., Block M.L., Liu Y., Breese G.R., Hong J.S., Knapp D.J., Crews F.T. (2007). Systemic LPS causes chronic neuroinflammation and progressive neurodegeneration. Glia.

[40] You M., Miao Z., Sienkiewicz O., Jiang X., Zhao X., Hu F. (2020). 10-Hydroxydecanoic acid inhibits LPS-induced inflammation by targeting p53 in microglial cells. Int. Immunopharmacol..

[41] Colonna M., Butovsky O. (2017). Microglia Function in the Central Nervous System During Health and Neurodegeneration. Annu. Rev. Immunol..

[42] Badoer E. (2010). Microglia: Activation in acute and chronic inflammatory states and in response to cardiovascular dysfunction. Int. J. Biochem. Cell Biol..

[43] You M., Wang K., Pan Y., Tao L., Ma Q., Zhang G., Hu F. (2022). Combined royal jelly 10-hydroxydecanoic acid and aspirin has a synergistic effect against memory deficit and neuroinflammation. Food Funct..

[44] Saleh S.R., Agwah R.G., Elblehi S.S., Ghareeb A.Z., Ghareeb D.A., Maher A.M. (2025). Combination of 10-hydroxy-decanoic acid and ZnO nanoparticles abrogates lead acetate-induced nephrotoxicity in rats: Targeting oxidative stress and inflammatory signalling. BMC Pharmacol. Toxicol..

[45] Maher A.M., Elsanosy G.A., Ghareeb D.A., Elblehi S.S., Saleh S.R. (2024). 10-Hydroxy Decanoic Acid and Zinc Oxide Nanoparticles Retrieve Nrf2/HO-1 and Caspase-3/Bax/Bcl-2 Signaling in Lead-Induced Testicular Toxicity. Biol. Trace Elem. Res..

[46] Ornellas-Garcia U., Cuervo P., Ribeiro-Gomes F.L. (2023). Malaria and Leishmaniasis: Updates on co-infection. Front. Immunol..

[47] Alkhaibari A.M., Alanazi A.D. (2022). Insecticidal, Antimalarial, and Antileishmanial Effects of Royal Jelly and Its ThreeMain Fatty Acids, trans-10-Hydroxy-2-decenoic Acid, 10-Hydroxydecanoic Acid, and Sebacic Acid. Evid. Based Complement. Alternat. Med..

[48] Komalamisra N., Trongtokit Y., Rongsriyam Y., Apiwathnasorn C. (2005). Screening for larvicidal activity in some Thai plants against four mosquito vector species. Southeast Asian J. Trop. Med. Public Health.

[49] Ravi Kiran S., Bhavani K., Sita Devi P., Rajeswara Rao B.R., Janardhan Reddy K. (2006). Composition and larvicidal activity of leaves and stem essential oils of Chloroxylon swietenia DC against Aedes aegypti and Anopheles stephensi. Bioresour. Technol..

[50] Lombardo D., Kiselev M.A., Magazù S., Calandra P. (2015). Amphiphiles Self-Assembly: Basic Concepts and Future Perspectives of Supramolecular Approaches. Adv. Cond. Matter Phys..

[51] Gasbarri C., Angelini G. (2014). Spectroscopic investigation of fluorinated phenols as pH-sensitive probes in mixed liposomal systems. RSC Adv..

[52] Wang C., Wang Z., Zhang X. (2012). Amphiphilic Building Blocks for Self-Assembly: From Amphiphiles to Supra-amphiphiles. Acc. Chem. Res..

[53] De Maria P., Fontana A., Siani G., D’Aurizio E., Cerichelli G., Chiarini M., Angelini G., Gasbarri C. (2011). Synthesis and aggregation behaviour of a new sultaine surfactant. Coll. Surf. B Biointerfaces.

[54] Mondal B., Gupta V.K., Hansda B., Bhoumik A., Mondal T., Majumder H.K., Edwards-Gayle C.J.C., Hamley I.W., Jaisankar P., Banerjee A. (2022). Amino acid containing amphiphilic hydrogelators with antibacterial and antiparasitic activities. Soft Matter.

[55] Cross E.R., Schweins R., Coulter S.M., Fuentes-Caparro A.M., McAulay K., Schweins R., Laverty G., Adams D.J. (2020). Tuning the antimicrobial activity of low molecular weight hydrogels using dopamine autoxidation. Chem. Commun..

[56] Civelek I. (2022). Biological activities of royal jelly: A mini-review. Anatol. J. Biol..

[57] Angioi R., Morrin A., White B. (2021). The rediscovery of honey for skin repair: Recent advances in mechanisms for honey-mediated wound healing and scaffolded application Techniques. Appl. Sci..

[58] Tan D., Zhu W., Liu L., Pan Y. (2023). In situ formed scaffold with royal jelly-derived extracellular vesicles for wound healing. Theranostics.

[59] Hong S., Baravkar S.B., Lu Y., Masoud A.R., Zhao Q., Zhou W. (2025). Molecular Modification of Queen Bee Acid and 10-Hydroxydecanoic Acid with Specific Tripeptides: Rational Design, Organic Synthesis, and Assessment for Prohealing and Antimicrobial Hydrogel Properties. Molecules.

[60] Cragg G.M., Pezzuto J.M. (2016). Natural Products as a Vital Source for the Discovery of Cancer Chemotherapeutic and Chemopreventive Agents. Med. Princ. Pract..

[61] Demain A.L., Vaishnav P. (2011). Natural products for cancer chemotherapy. Microb. Biotechnol..

[62] Boretti A. (2022). Natural Products as Cancer Chemo-Preventive Agents: Where We Stand. Nat. Prod. Commun..

[63] dos Santos France F.A., Maeda D.K., Rodrigues A.B., Ono M., Marchetti F.L.N., Marchetti M.M., Martins A.C.F., da Silva Gomes R., Rainho C.A. (2024). Exploring fatty acids from royal jelly as a source of histone deacetylase inhibitors: From the hive to applications in human well-being and health. Epigenetics.

[64] DEWS Epidemiology (2007). The epidemiology of dry eye disease: Report of the epidemiology subcommittee of the international dry eye WorkShop. Ocul. Surf..

[65] Stapleton F., Alves M., Bunya V.Y., Jalbert I., Lekhanont K., Malet F., Na K.S., Schaumberg D., Uchino M., Vehof J. (2017). TFOS DEWS II Epidemiology Report. Ocul. Surf..

[66] Bu J., Liu Y., Zhang R., Lin S., Zhuang J., Sun L., Zhang L., He H., Zong R., Wu Y. (2024). Potential New Target for Dry Eye Disease—Oxidative Stress. Antioxidants.

[67] Inoue S., Kawashima M., Hisamura R., Imada T., Izuta Y., Nakamura S., Ito M., Tsubota K. (2017). Clinical evaluation of a royal jelly supplementation for the restoration of dry eye: A prospective randomized double blind placebo controlled study and an experimental mouse model. PLoS ONE.

[68] Yamaga M., Imada T., Tani H., Nakamura S., Yamaki A., Tsubota K. (2021). Acetylcholine and Royal Jelly fatty acids combinations as potential dry eye treatment components in mice. Nutrients.

[69] Hansen M.B. (2003). Neurohumoral control of gastrointestinal motility. Physiol. Res..

[70] Miyauchi-Wakuda S., Kagota S., Maruyama-Fumoto K., Wakuda H., Yamada S., Shinozuka K. (2019). Effect of royal jelly on mouse isolated ileum and gastrointestinal motility. J. Med. Food.

[71] Moriyama T., Yanagihara M., Yano E., Kimura G., Seishima M., Tani H., Kanno T., Nakamura-Hirota T., Hashimoto K., Tatefuji T. (2013). Hypoallergenicity and immunological characterization of enzyme-treated royal jelly from Apis mellifera. Biosci. Biotechnol. Biochem..

[72] Cornara L., Biagi M., Xiao J., Burlando B. (2017). Therapeutic properties of bioactive compounds from different honeybee products. Front. Pharmacol..

[73] Koya-Miyata S., Okamoto I., Ushio S., Iwaki K., Ikeda M., Kurimoto M. (2004). Identification of a collagen production-promoting factor from an extract of royal jelly and its possible mechanism. Biosci. Biotechnol. Biochem..

